# Comparative proteomic data of M13SV1 human breast epithelial cells and their tumorigenic variants under treatment with estrogenic compounds

**DOI:** 10.1016/j.dib.2016.05.052

**Published:** 2016-05-30

**Authors:** Albert Braeuning, Claudia Schmidt, Axel Oberemm, Alfonso Lampen

**Affiliations:** German Federal Institute for Risk Assessment, Department Food Safety, Max-Dohrn-Str. 8-10, 10589 Berlin, Germany

## Abstract

Data from a comparative proteomic analysis of three human breast epithelial cell lines are presented. M13SV1 cells and their tumorigenic derivatives M13SV1-R2-2 and M13SV1-R2-N1 were used. Proteomic data were obtained using 2-dimensional gel electrophoresis and subsequent identification of proteins by MALDI-TOF mass spectrometry. In a second experiment, the three cell lines were treated with different concentrations of the estrogenic compounds β-estradiol or genistein and alterations in protein expression were monitored by 2-dimensional gel electrophoresis and MALDI-TOF mass spectrometry. Presented data provide a comprehensive overview of proteomic differences between the three cell lines and their response to estrogenic stimulation.

**Specifications Table**

**Table:** 

Subject area	*Biology*
More specific subject area	*Cell biology / Toxicology / Proteomics*
Type of data	*Figures, protein expression tables*
How data was acquired	*Cell culture, 2-dimensional gel electrophoresis, MALDI-TOF mass spectrometric identification of proteins*
Data format	*Normalized data.*
Experimental factors	*Cell line identity, treatment with genistein or β-estradiol, 2D/MS proteome analysis*
Experimental features	*M13SV1 human breast epithelial cells and their tumorigenic variants M13SV1-R2-2 and M13SV1-R2-N1 were treated with different concentrations of genistein or β-estradiol. Protein extracts were separated by 2-dimensional gel electrophoresis. Following identification of significantly altered protein spots, spots were picked and identified using MALDI-TOF mass spectrometry.*
Data source location	*German Federal Institute for Risk Assessment, Berlin, Germany*
Data accessibility	*Data is with this article.*

**Value of the data**

• Differences between M13SV1 human breast epithelial cells and their tumorigenic variants M13SV1-R2-2 and M13SV1-R2-N1 were identified by comprehensive proteomic analysis
• Quantitative proteomic responses of the three cell lines to the estrogenic compounds genistein and β-estradiol were identified
• Data can help to elucidate biological mechanisms of tumorigenicity and the molecular response of breast cancer cells to estrogenic substances.

## 1 Data

Proteomic profiles of the three cell lines M13SV1 human breast epithelial cells [1] and their tumorigenic variants M13SV1-R2-2 and M13SV1-R2-N1 [2] were obtained by 2-dimensional gel electrophoresis followed by identification of significantly differentially expressed protein spots by MALDI/TOF mass spectrometry. In addition, the proteomic responses of the three cell lines to treatment with different concentrations of the estrogenic compounds genistein and β-estradiol were analyzed.

**Fig. 1:**
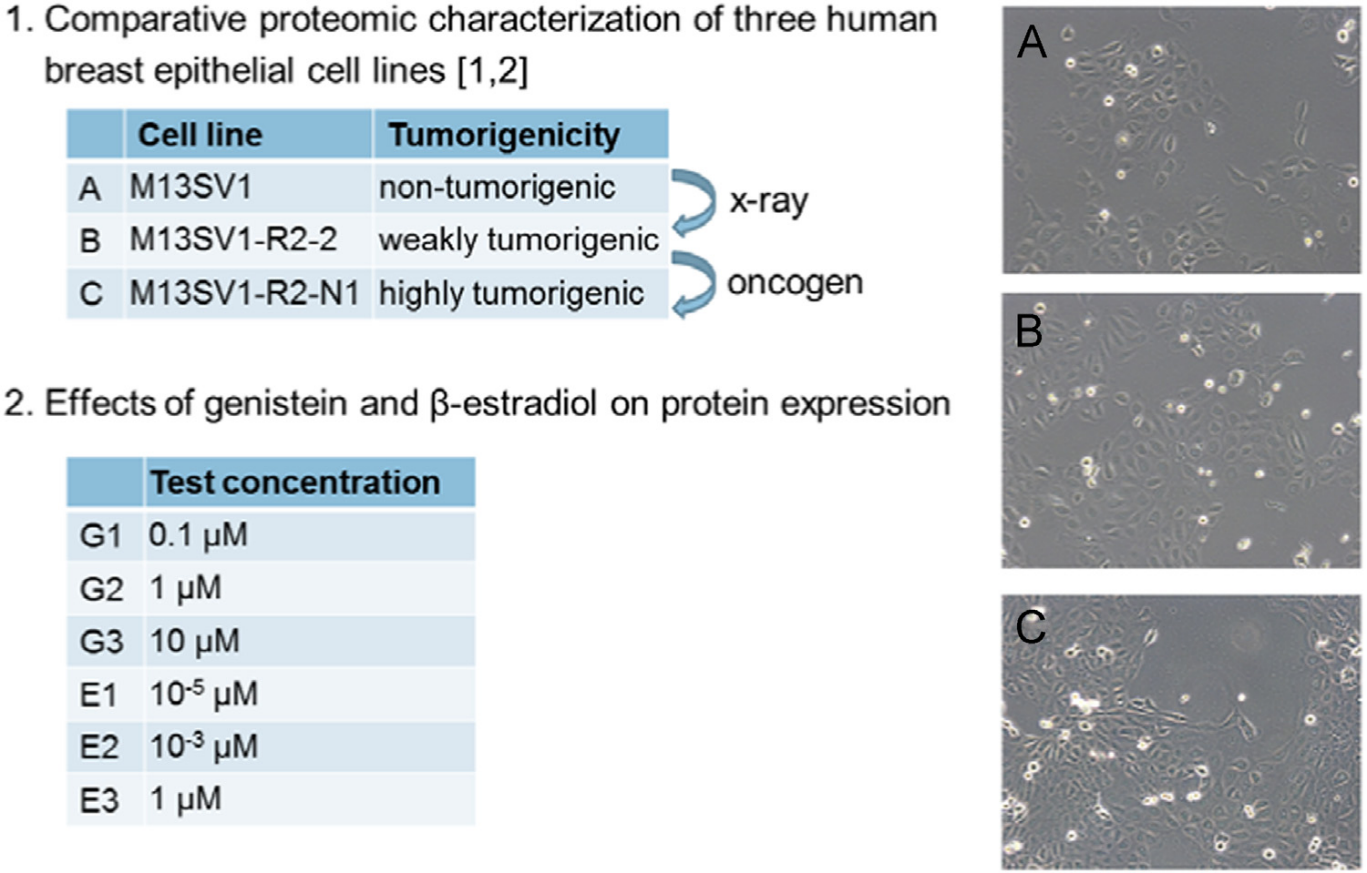
Overview of cell lines and treatment regimen used in the study. Images show representative photographs of the three cell lines. For details, please refer to the text. Data contained in this paper comprise a comparative proteomic characterization of the cell lines (A–C) as well as an analysis of protein deregulation following treatment with the estrogenic substances genistein (G) or β-estradiol (E). For generation of the cell lines with different tumorigenicity, please refer to published literature [1], [2].

Data are presented in form of an Excel spreadsheet (supplementary file) containing information about the identity of the proteins, fold difference in abundance between different cell lines or different treatments, and corresponding *p* values. Table 1 of the supplementary file contains the data of a comparison of cell lines, i.e. proteins differentially expressed in the tumorigenic cell lines M13SV1-R2-2 (termed “B” throughout the supplementary file) and M13SV1-R2-N1 (termed “C”), as compared to the non-tumorigenic line M13SV1 (termed “A”). Tables 2-4 of the supplementary file contain the data of proteins de-regulated in cell lines A-C following treatment with genistein (0.1 µM, 1 µM, 10 µM; termed “G1-G3”) or β-estradiol (10^−5^ µM, 10^−3^ µM, 1 µM; termed “E1-E3”). A legend with explanations is contained in the last tab, termed “explanations”. Please see also Fig. 1 for an overview of cell lines and treatment regimen.

## 2 Experimental design, materials and methods

### 2.1 Cell culture and treatment

Human breast epithelial cells from line M13SV1 [1] and their tumorigenic derivatives M13SV1-R2-2 and M13SV1-R2-N1 [2] were cultured at 37 °C and 5% CO_2_ in Michigan-State-University-1 (MSU-1) medium (Biochrom, Berlin, Germany), supplemented with 5% charcoal-stripped fetal calf serum and antibiotics (PAA, Pasching, Austria). Cells were treated at ~80% confluency with different concentrations of genistein or β-estradiol for 24 h (see also Fig. 1). Controls were incubated with 0.1% (v/v) EtOH as a solvent control. For harvesting, cells were washed three times with pre-chilled phosphate-buffered saline and subsequently lysed with lysis buffer (7 M urea, 2 M thiourea, 2% pharmalyte (pH3-10), 0.7% spermine, 1.2% destreak reagent, 4% chaps, serdolit mb-1, and proteinase inhibitor) [3] under shaking for 30 min following centrifugation at 100,000*g* for 60 min at 15 °C. Supernatant was stored at −80 °C for further analysis. Protein content was determined by use of the Bradford assay.

### 2.2 Two-dimensional gel electrophoresis

Two-dimensional gel electrophoresis has been described in detail in a previous paper [3]. For details on the experimental setup, please refer to the latter publication. In brief, isoelectric focusing using IPG strips (pH 3–10) was followed by SDS-PAGE in a 12.5% acrylamide gel. Spots were stained with Tris Ruthenium II. Four gels (technical replicates) were run per sample, in biological triplicates for each experimental condition. Protein spots were identified and quantified using a VersaDoc imaging system and ProteinMine 1.6.1 software.

### 2.3 Statistical evaluation of gels

Data analysis and statistical evaluation of 2D gels using the software R has been described in detail in a previous paper [3]. For details on the experimental setup, please refer to the latter publication. A normalization of 2-DE gel images was performed using the total spot volume, related to an anchor template gel image as reference. Spot intensity values, given as sum of pixel intensities of each spot area, were used as a measure for protein spot quantification. Only spots quantified in at least two out of three biological and two out of four technical replicates were considered for statistical analysis. Comparison of treatment groups was done using a two-sided Wilcoxon rank sum test and statistical significance was assumed at *p*<0.05). A cutoff of │log_2_ ratio│≥0.3 for up- or downregulation of individual protein spots was applied.

### 2.4 Spot picking and in-gel digestion

**Fig. 2:**
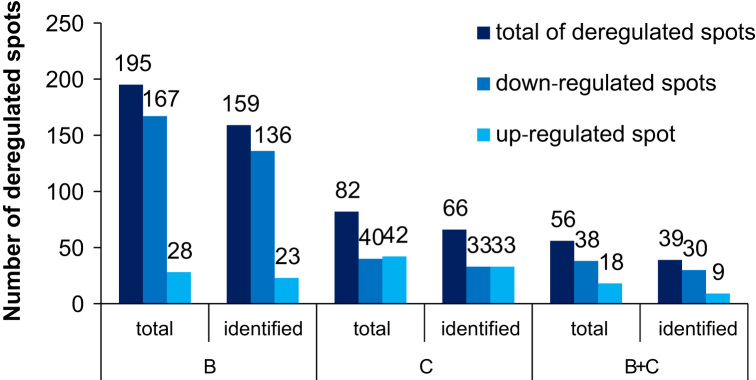
Overview of the numbers of up- or downregulated protein spots in the cell line comparison experiment. B (left) and C (middle) indicate the numbers of differentially expressed spots in cell lines B or C, respectively, as compared to cell line A. B+C (right) indicates the numbers of spots differentially expressed between cell line A and both derivatives, cell lines B and C. For comparison, data on total spots and the numbers of spots identified by mass spectrometry are shown.

**Fig. 3:**
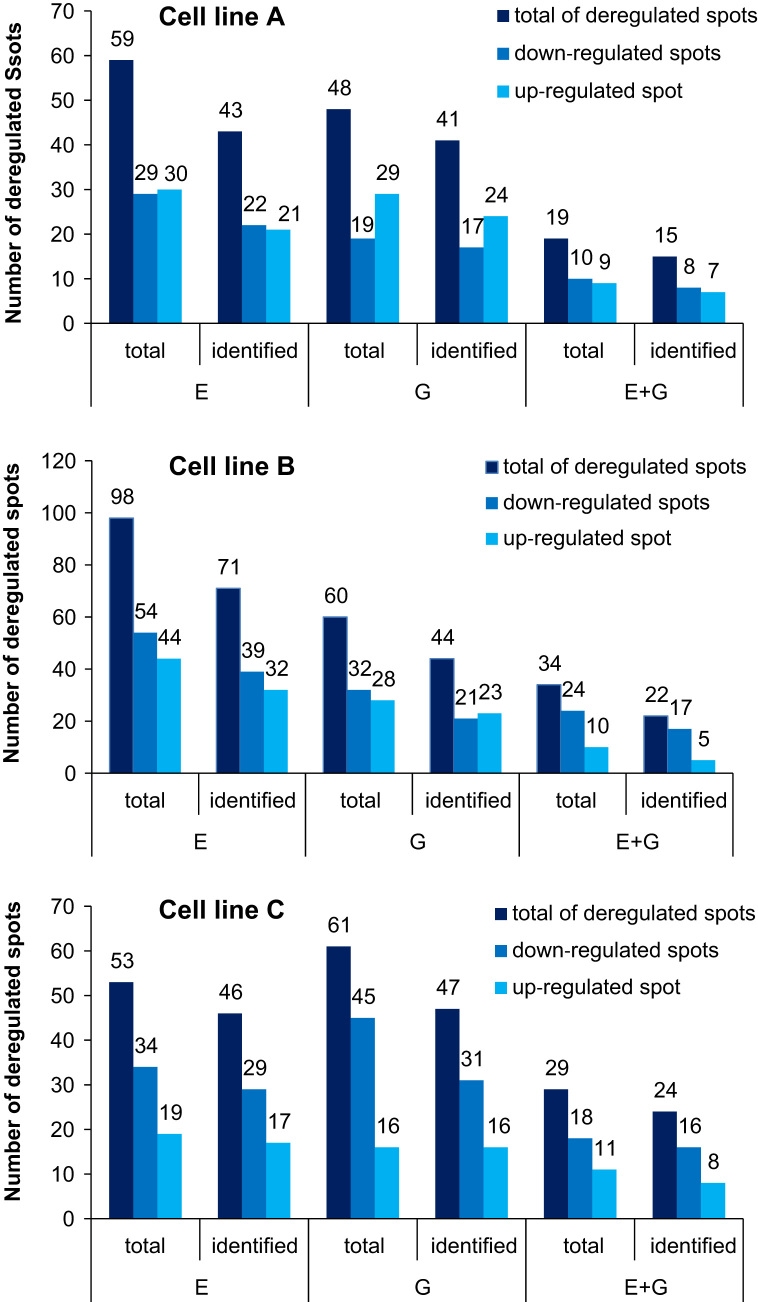
Overview of the numbers of up- or downregulated protein spots in the estrogenic cell treatment experiment. The sum of spots deregulated in response to cell treatment with different concentrations of β-estradiol (E,) genistein (G), or both (E+G) is shown separately for the three cell lines. For comparison, data on total spots and the numbers of spots identified by mass spectrometry are shown.

Robot-assisted spot picking and tryptic in-gel digestion methodology has been described in detail in a previous paper [3]. For details on the experimental setup, please refer to the latter publication. Approximately 3000 spots were identified per gel. For an overview of the number of deregulated protein spots in the cell line comparison and the estrogenic cell treatment experiment, please refer to Fig. 2 and Fig. 3.

### 2.5 Mass spectrometry

Mass spectrometric analyses were used for protein identification, whereas protein quantification was based on spot intensity evaluation of 2D gel electrophoresis (see also section 2.2). Protein identification by mass spectrometry has been described in detail in a previous paper [3]. For details on the experimental setup, please refer to the latter publication. In brief, digested proteins were manually spotted on an AnchorChip Target (800/384, Bruker) and matrix solution was added. An Ultraflex II mass spectrometer (Bruker) with smartbeam laser was used for matrix-assisted laser desorption/ionization mass spectrometry (MALDI-MS) analysis with a mass range of 500–5000 Da. For more details on mass spectrometer settings, please refer to [3]. Flex Control and Flex Analysis software (version 3.0, Bruker) were used to process MS and MS/MS spectra. Protein identification was performed using Biotools software (version 3.2, Bruker) with Mascot search using the SwissProt database (accessed between January 09th and June 17th, releases 2015_01 −2016_06; taxonomy: homo sapiens). For each protein to identify, two protein spots were digested and measured independently using peptide mass fingerprinting and MS/MS, giving 4 results. Only proteins identified with a minimum of 2 hits were considered valid. The column header “hits” in the Supplementary file shows the number of identifications for the two spot measurements. Sequence coverage of each identified protein is also indicated in the Supplementary file.

## Footnotes

Appendix A Supplementary data associated with this article can be found in the online version at doi:10.1016/j.dib.2016.05.052.
