# Supplementary material for: Comparative proteomic data of M13SV1 human breast epithelial cells and their tumorigenic variants under treatment with estrogenic compounds
Source: Data Brief. 2016 May 30;8:329–33. doi: 10.1016/j.dib.2016.05.052 (PMC4909722; doi:10.1016/j.dib.2016.05.052)
Supplement: Supplementary file 1 — Supplementary material [file mmc1.docx]

*Conflict of interest declaration*

**Manuscript title: Comparative proteomic data of M13SV1 breast cancer cells and their tumorigenic variants under treatment with estrogenic compounds**

**Authors: Albert Braeuning, Claudia Schmidt, Axel Oberemm, Alfonso Lampen**

**Affiliations: Federal Institute for Risk Assessment, Dept. Food Safety, Max-Dohrn-Str. 8-10, 10589 Berlin, Germany**

**Contact email: Albert.Braeuning@bfr.bund.de**

All authors declare that there are no conflicts of interest.

Signed May 12, 2016, on behalf of all co-authors

Albert Braeuning

p.s.: This document is submitted to the journal due to the fact that the link to the conflict of interest form in the online submission system on the journal’s web page is not functional.
